# A Case of Pneumonia, Succeeded by Purpura Hemorrhagica and Abscess

**Published:** 1846-06

**Authors:** W. H. Stilwell

**Affiliations:** Madison county, Tennessee


Art. IV.—*A Case of Pneumonia, succeeded by Purpura Hemorrhagica and Abscess.* By W. H. Stilwell, M. D., of Madison county, Tennessee.

J. B———, a laborer, aged 17 years, had an attack of pneumonia four years ago; was seized 9th January last with chill followed by fever, pain in head, cough, with free expectoration of bloody mucus. Pain in left side of the chest about the nipple constant and much aggravated by coughing. I was called on the 10th, and found the pulse 90, small and compressible; surface moderately warm and pale, except the 'circumscribed blush' on the cheeks; tongue moist and slightly coated with whitish yellow fur, edges red; thirst considerable; no nausea; bowels costive. Every act of coughing brought up a mouthful of thin, viscid, bloody mucus. Respiration 40, and anxious. I prescribed the following: Antim. tart. grs.4, aquæ ℥vi, a tablespoonful to be taken every hour until it produced vomiting. Ten grains of blue mass were also exhibited in divided doses at the same time. Scarification and cups were applied over the region of the inflamed lung.

11th. Medicine vomited the patient several times, and produced hypercatharsis; evacuations bilious; pain in head relieved, that in the chest mitigated a little. Temperature the same; cough less frequent, but painful; sputa as before.

Ordered a large blister to left side of chest; calomel 1 grain, opium ½grain, every three hours. Continue the antimonial solution in nauseating doses.

12th. Medicines had been regularly taken; tolerance of antimony established. Blister drew well, and pain much relieved, but migrating into sternal region; cough and sputa the same.

It would be unnecessarily tedious to detail the daily progress of this case throughout. The tart. antim., calomel, and occasionally opium, with repeated blistering, were persevered in with but slight alteration of the patient's condition until about the 9th day, when great apparent amendment began to be visible. There was then gentle diaphoresis; pulse 85, and weak; pain slight; cough occasional; sputa yellow, opaque, without blood; tongue cleaning; no thirst, and appetite returning. In short, on the 23d my visits were discontinued, under the belief that he was convalescent and out of danger.

On the 25th I was again sent for, and found him with haggard countenance; eyes dull; skin natural, moist; pulse scarcely perceptible, and about 110; no pectoral pain; cough almost gone; but he was bleeding slowly from the nose; had a constant desire to urinate, with excrutiating pain in the region of the bladder, and passed from a gill to half a pint of almost pure blood at each evacuation of that organ. His breast and extremities were punctuated with dark red specks, (petechiæ) the size of a pin's head, without elevation at first; on detachment of the cutis, an escape of blood took place sufficient to stain the sheets in spots. Blood was also oozing from the surface of the last blister. The bowels were in a natural condition; the patient was without appetite. Prescribed alum and opium at the moment, and afterwards pills of acetate of lead 1gr., opium ½gr.; one to be taken every two hours.

26th. Patient in the same state. Directed port wine to be given freely as a drink and in his food. In addition to the above medicines, gave 12 drops tinct. mur. iron every two hours; lead water to be applied to the hypogastrium.

27th. Patient better; continued the same medicines, and in a few days the hemorrhage ceased entirely; his appetite returned; his pulse became fuller, but continued too frequent; hectic flushes and some nocturnal sweating were present; bowels confined four or five days from the opium. Pain was again felt in the left side of the chest two inches above the nipple, attended by dullness on percussion, a total absence of respiratory murmur, and pectoriloquy. Cough became in a short time paroxysmal and convulsive. This condition of things lasted until the morning of the 15th of February, when, while struggling with the most violent fit of coughing he had ever experienced, the abscess burst, and he was nearly suffocated by the discharge from the lungs of half a chamberpotful of a yellowish purulent fluid, of a pungent, penetrating odor. He continued to expectorate for several weeks; the matter becoming more consistent and daily diminishing in quantity, while his general condition improved rapidly. He is now free from all complaint; no medicine whatever was taken after the discharge of the abscess.

Pneumonia is more or less prevalent in all the western counties of this State, during the latter autumnal and the winter months. Indeed, a gradual transition from autumnal bilious remittent fever, to pneumonia, is often observed; the first examples of the latter partaking always partially of the bilious form. Later, the disease assumes more of the inflammatory character; although it seems to be generally admitted that there exists a material difference between the disease as it occurs here and in more northern latitudes. Here there is perhaps more hepatic derangement, and less tolerance of a vigorous antiphlogistic treatment than in colder regions. True pleurisies are rarely seen. It is observed that men are most liable to attacks of pneumonia, in consequence perhaps of their greater exposure, the largest proportion of cases occurring among the laboring classes. The period of life most liable to attacks of the disease is from fifteen to thirty years.

During the past winter there were about the usual number of pneumonic cases, and as a general remark they presented the bilious appearances.

The above case, and a few others which occurred in the practice of a neighboring physician, seem to constitute exceptions. I did not see the others alluded to; they all occurred in one family, and were described as bearing a close resemblance to the one detailed above; the chief difference consisting in an earlier development and greater intensity of the typhoid symptoms, and also in their proving fatal in about half the instances.

Such cases as that of J. B. are not of frequent occurrence, if I may judge from my own observation. One strikingly analogous to it, however, in its origin, progress, and termination, is reported by Dr. L. P. Yandell in the Transylvania Journal of Medicine, (vol. I, page 237), the following extracts from which will be read, I think, with interest:

"Miss G., daughter of a respectable farmer in the vicinity of Murfreesborough, Tennessee, aged about eighteen years, of a stout frame, and robust habit, was attacked on the 15th day of January, 1827, with pleurisy, blended with peripneumonic symptoms. The attack being violent, an energetic practice, consisting of venesection, cathartics, and nauseating doses of antimony, was resorted to. It was found necessary to repeat the bleeding more than once, and calomel, in ten grain doses, was given for several nights in succession, followed by saline purgatives next day. Blisters and diaphoretics succeeded in due time, and the disease appeared to be yielding to the treatment.

"But on the fifth day of her indisposition, the patient experienced an attack of diarrhœa, by which she was alarmingly prostrated. It yielded, however, in a few days, to opium and acetate of lead, conjoind with the compound powder of ipecacuan; and by the use of the carbonate of ammonia she was again speedily convalescent.

"On the 30th we heard from her, at which time she was considered cured. We saw her, incidentally, on the 31st; she was engaged in light manual labor, but she still experienced some pain in the breast, slight cough, and dyspnœa. Believing that some vestige of the disease, in the shape of sub-acute inflammation still remained, we ordered her a grain of calomel, with a fourth of a grain of opium every night on going to bed, with a view of producing an alterative effect.

"We left our patient hoping to hear no more of the case. But the worst was yet to follow. On the 5th of February she was attacked with hemorrhage from the nose and gums, which resisting all the domestic remedies of the family, caused us to be called on the night of the 11th. When we arrived at her bed-side we found her prostrated to the lowest point; her countenance exsanguineous and sunken; her voice weak and tremulous; pulse extremely frequent and scarcely perceptible; the hemorrhage from her nose and gums still in progress. In the course of the day, upon a slight muscular effort, she had once experienced syncope.

"Upon examination we found the extremities covered with petechiæ, and were informed that they existed on other parts of the body. They were to be seen lining the tongue, gums and fauces. Blood came, occasionally, from the ears, and from the nostrils it fell in drops without intermission. It was ejected from the mouth with the saliva.

"The whole class of vegetable and mineral styptics had been gone over without effect—the bleeding continuing, and the patient in the most imminent peril.

"Without loss of time we commenced with bark and the sulphuric acid, in doses as large as her stomach would bear. A gargle made of borax, and cold cloths to the back of her neck, were conjoined, with temporary advantage.

"In a few days, under this course, the hemorrhage ceased; the vital powers rallied; the petechial spots disappeared; and considering her extreme debility, she enjoyed a rapid convalescence, and is now in the enjoyment of good health."

In this case no abscess formed in the lungs; but in every other particular it bears a strong resemblance to the one the history of which I have given. I have been disposed to ask, in reviewing the case of J. B., whether the disease with which he suffered differs from the "*pneumonia typhodes*" of the eastern and middle States; and whether it was not, from the beginning, a typhoid disease? I have also sometimes asked myself, whether the free use of the tartarized antimony had any agency in inducing the hemorrhage, and other secondary symptoms? I find that Dr. Yandell, too, was inclined to look to the treatment for the origin of the subsequent evils in his case. "Cathartics," he says, "were possibly administered too freely in the acute stage of the complaint. The diarrhœa, with the consequent extreme prostration, was probably produced by this cause." He supposes it "possible that the calomel was the cause of the purpura," although he insists, on good grounds, that the mercurial was indicated in the inflamed condition of the lungs. I have supposed that the more liberal use of calomel, in my case, might have prevented the formation of the abscess. Tonics, in Dr. Y.'s case were beneficial. Would they, in mine, have saved the patient from the typhoid symptoms, if they had been seasonably interposed? The whole subject of purpura, its cause, pathology, and best treatment, is a most obscure one, and the discrepancy of opinion concerning it as great as it was half a century ago.

March 10th, 1846.

